# Contribution of (non-)starch polysaccharides to distinctive sensory perception in beer – Significance of their physical and friction characteristics

**DOI:** 10.1016/j.crfs.2025.101118

**Published:** 2025-06-18

**Authors:** Rolando Cesar Moreno Ravelo, Christoph Neugrodda, Martina Gastl, Thomas Becker

**Affiliations:** a Technical University of Munich, Institute of Brewing and Beverage Technology, Group Raw Material Based Brewing and Beverage Technology, Weihenstephaner Steig 20, 85354, Freising, Germany; b Technical University Munich, Research Center Weihenstephan for Brewing and Food Quality, Alte Akademie 3, 85354, Freising, Germany

**Keywords:** Soft tribology, Molar mass, Beer, Palate fullness, Mouthfeel, Conformation, Polysaccharides

## Abstract

The *palate fullness* (body) and *mouthfeel* are important sensory attributes influencing the consumers’ beer preference. Among the several parameters affecting these attributes, recent research suggests that the physical characteristics (molar mass and conformation) of starch (dextrins) and non-starch polysaccharides (arabinoxylans and β-glucans) play a critical role. However, the lack of information regarding the physical state of these polysaccharides in beer hinders the sensory evaluation of each component. A method was recently introduced to isolate and solely characterize the molar mass and conformation of beer's starch and non-starch polysaccharides in solution by AF4-MALS-DRI. Therefore, this research evaluated the relationship between the molar mass and conformation of arabinoxylans, β-glucans, and dextrins with the *palate fullness* and *mouthfeel* sensorial perception. Additionally, friction, assessed by soft tribology, was analyzed as a triggering mechanism during oral processing. Grains from different sources modified at a low level (steeping degree as parameter) were used to produce bottom-fermented beers with diverse physical characteristics. Regarding the friction response, the variation of correlation behavior at different sliding velocities suggests that the human sensory panel might perceive the *palate fullness* and *mouthfeel* at different stages during oral processing. The multivariate analysis suggests that the conformation ratio, $r_{rms}/r_{hyd}$, from each polysaccharide triggers a distinctive sensory response, being dextrins related to *palate fullness* while arabinoxylans and β-glucans to *mouthfeel*. Furthermore, the beer sample substituted with barley modified at a low level presented an increase of branched dextrins in comparison to the (unsubstituted) control, which was related to its increase in *palate fullness intensity*.

## Highlights

• Using low modified malt changes the physical characteristics of beer polysaccharides.
• The conformation of each polysaccharide influenced a distinctive sensory attribute.
• Micro-gel and swollen β-glucans were correlated to a slimy mouthfeel descriptor.
• Branched DXs were appointed to increase palate fullness intensity.
• Soft tribology suggests palate fullness and mouthfeel are perceived at different stages in the mouth.

## Graphical abstract

**Figure:**
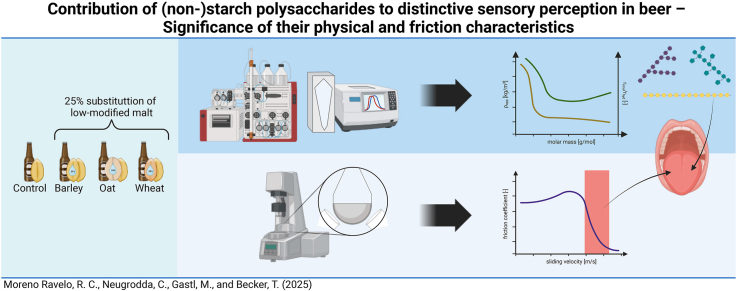

## 1 Introduction

Cereal-based beverages are some of the most consumed products in the world. Among their multiple sensory attributes, *palate fullness* (*PF;* weight resistance to flow often interchangeable with *body* (Langstaff and Lewis, 1993; Krebs et al., 2021)) and *mouthfeel (mixed response of physical or chemical stimulus in the mouth* (DIN EN ISO - 5492, 2009) are relevant for consumer preference (Agorastos et al., 2023). Several studies suggest that chemical parameters (e.g., concentration) influence these sensory attributes, as starch and non-starch polysaccharides ((N)SPs) are relevant (Rübsam et al., 2013a; Langstaff et al., 1991a; Langenaeken et al., 2020; Michiels et al., 2025). Nevertheless, the multivariate nature of the beer and non-alcoholic beers (NAB) hinders elucidating the specific sensory contribution from these (N)SPs. On the other hand, soft tribology has emerged as a tool to analyze food products from the perspective of oral (drinking) processing. Previous research showed the influence of raw material modification on the soft tribology responses, which was attributed to the molar mass and conformation of (N)SPs (Moreno Ravelo et al., 2025). However, the relationship of these modifications to the sensory characteristics of cereal-based beverages has not been investigated.

The physical characteristics (molar mass and conformation) and concentration of (N)SPs are important for their functionality in the brewing industry. For example, the β-glucans (BGs) concentration, which is a contributor to the products’ viscosity, has a recommended maximum concentration in barley malt of less than 350 mg/L (iso-65 °C (Back et al., 2019)) to prevent filtration difficulties (Kupetz et al., 2015). Furthermore, the (N)SPs bear sensory relevancy in cereal-based beverages. The arabinoxylans (AXs) and dextrins (DXs) have been appointed as positive contributors to *PF* (Rübsam et al., 2013a; Langenaeken et al., 2020) and BGs to the *slimy mouthfeel* descriptor (Krebs et al., 2019), with the concentration ratio of DXs and BGs being important (Moreno Ravelo et al., 2024a). Besides (N)SPs' concentration, their molar mass has also been reported as a positive contributor to *PF* and *mouthfeel* in beer and NABs (Langenaeken et al., 2020; Moreno Ravelo et al., 2024a; Krebs et al., 2020). For example, the combination of low and high molar mass DXs and AXs (respectively) increased the *PF* intensity of NAB (Michiels et al., 2025), while BGs in the range of 100–1000 kDa produced a more slimy NAB (Krebs et al., 2019). Nevertheless, the contribution from each (N)SPs to the sensory characteristics, with particular attention to which molar mass and conformation parameters are relevant, is yet unclear.

Tribology investigates lubrication, friction, and wear between interacting surfaces in relative motion (Stokes et al., 2013). An essential difference between tribology and rheology is that the former is a system property, which implies that the lubricant (food), the measurement system, and the surface material influence the friction response (Shewan et al., 2020). Thus, special attention is needed when comparing tribology data. The resulting friction coefficient (FC) is usually plotted as a function of the sliding velocity, which is referred to as the Stribeck curve. These curves can be further divided into different regimes depending on the film thickness between the surfaces, with the boundary and mixed regimes being relevant in food oral processing (Selway and Stokes, 2014) and cereal-based beverages such as beer and non-alcoholic beer (Fox et al., 2022). Although *Mouthfeel* descriptors are mainly correlated to food's tribology (e.g., creaminess (Laiho et al., 2017; Sonne et al., 2014), *slimy* (Krop et al., 2019), *pasty* (Krop et al., 2019), *smoothness* (Laiho et al., 2017; Nguyen et al., 2017), *mouth coating* (Laiho et al., 2017; Nguyen et al., 2017), and *astringent* (Fox et al., 2022; Brossard et al., 2016; Wang et al., 2020)), bulk characteristics such as thickness have also shown correlations to tribology measurements (Nguyen et al., 2017).

A dynamic velocity profile is exerted in the oral cavity during drinking, specifically when the tongue moves against the (hard) palate (Peng et al., 2000). The *ex-situ* replication of such a system is possible by using compliant surfaces to their biological counterpart. This strategy is called soft tribology, in which multiple compliant surfaces have been proposed to mimic the “in-mouth” oral processing. A pair of surfaces commonly used to mimic the oral cavity are the polydimethylsiloxane (PDMS) and steel ball for the tongue and palate, respectively (Biegler et al., 2016). Additionally, the tongue's velocity in the mouth during drinking (0.0032–0.32 m/s) can also be considered when analyzing the FC from the (physiological) oral processing perspective (Moreno Ravelo et al., 2025; Peng et al., 2000). Still, using soft tribology to further comprehend human sensory perception is yet to be explored in cereal-based beverages.

Although (N)SPs are relevant for the brewing industry, their physical contribution to sensory perception in beer has not been thoroughly investigated. The type of raw material and its modification degree are among the multiple parameters influencing the physical characteristics of (N)SPs. Previous research done with laboratory-produced wort demonstrated that the presence of higher molar masses and different conformations of these polysaccharides influences the lubrication (soft tribology) response, whose responses were altered through the usage of low modified malted grains (parameter steeping degree) (Moreno Ravelo et al., 2025). However, it is not known if these changes also influence the sensory characteristics of beer, specifically the *PF* and *mouthfeel*. Therefore, this work aimed to study the changes in beer perception by altering the physical characteristics of (N)SPs, analyzed by a human sensory panel and soft tribology. We hypothesize that the physical characteristics of each (N)SP trigger a distinct sensory response (*PF* or *mouthfeel*). Likewise, these perceived sensory differences (from a human sensory panel) can also be analyzed with soft tribology. Four beers were produced in triplicate on a small-scale (8 L) automated system. 25 % of the grain bill was substituted with low modified grains from different sources to alter the high molar mass responses of (N)SPs. A standard brewing (barley) malt was used to complete the rest of the grain bill and to produce an unsubstituted control sample. The high molar mass (N)SPs modification was confirmed by isolating and characterizing these polysaccharides according to a published methodology (Moreno Ravelo et al., 2024b). A human sensory panel assessed different *PF* and *mouthfeel* attributes, followed by the soft tribology determination with a ball-on-three-pin system (Moreno Ravelo et al., 2025). At last, multivariate data analysis was used to comprehend the relationship among (N)SPs, soft tribology, and sensory characteristics of the beers.

## 2 Materials and methods

### 2.1 Standardized beer production on small (8 L) scale

Beers were produced by using malts from different sources, modified on a low level (39 % steeping degree). These grains were chosen as they contributed (N)SPs with higher molar masses and diversity of molecular structures compared to their high level of modification counterpart (45 % steeping degree) on a laboratory scale (Moreno Ravelo et al., 2025). The detailed malting procedure can be observed elsewhere (Moreno Ravelo et al., 2025). However, the standard malt analysis according to iso-65 °C mash (R-207.00.002) based on MEBAK (Jacob, 2013a) is observed in the supplementary data (Table A.1): extract (R-205.01.080 [2016–03]), viscosity (R-205.10.282 [2016–03]), BGs content (R-205.15.174 [2016–03]), soluble nitrogen (R-205.11.030 [2016–03]), free-amino nitrogen (R-205.14.111 [2016–03]), final degree of attenuation (R-205.17.080 [2016–03]), and friability (R-200.14.011 [2016–03].

Low modified barley (B39), oat (O39), and wheat (W39) malts substituted 25 % of a standard pilsner malt (Weyermann, Bamberg, Germany). A control sample brewed with 100 % of the standard pilsner malt was also produced. The same batch of standard pilsner malt was used on all the beers. The chosen characteristics of the (lager) beer were an original gravity of 11 % w/w, 5 vol% ethanol, and 20 bitter units. The beers were produced with an 8 L automated small-scale brewery (Joh. Albrecht Brautechnik JBT GmbH, Germany). 1.5 kg of malt was milled in a 2-roll dry malt mill (MIAG, Germany). Infusion mashing was used starting at 62 °C for 30 min, followed by a temperature increase rate of 1 °C per minute until reaching 72 °C, which was held for 30 min. The mashing temperature was increased to 78 °C and was retained for 10 min before lautering. Hop CO_2_ extract (Perle variety, HVG, Germany) was added during wort boiling (1 h). The whirlpool rest was 15 min, followed by wort cooling (12 °C) and yeast pitching (Saflager W-34/70 by Fermentis, France, rehydrated and allowed to revive in first wort). Fermentation lasted 5 days at 12 °C until the extract value dropped below 3.5 % w/w. Maturation of green beer occurred at 16 °C until it reached a total diacetyl value below 0.1 mg/L (analyzed according to MEBAK 2.21.5.1). The beers were stored at 0 °C for four weeks before filtration (three filter layers, K150, Pall Corporation, Germany), and bottled in 0.33 L brown longneck bottles under CO_2_ and 4 °C. Due to the limitations of the number of fermentation tanks, there was a three-week difference between the first and last beer produced. The beers were produced in triplicate.

Different methods assessed the chemical characteristics of the beers. The pH, alcohol, original gravity, apparent extract, real extract, viscosity, viscosity adjusted (adj.) to 8.6 %, apparent attenuation, and real attenuation were measured with Anton Paar Alcolyzer in combination with a density meter (DMA 4500, Anton Paar, Germany) and a rolling and falling ball viscometer (Lovis, 2000 ME, Anton Paar, Germany). The final degree of attenuation (B-420.36.530 [2020-10]), soluble nitrogen (B-400.07.003 [2020-10]), iso-alpha acids (B-400.19.131 [2020-10]), bitter units (B-400.17.110 [2024-05]), total polyphenols (B-590.41.111 [2020-10]), were analyzed by following MEBAK-guidelines (Jacob, 2013b). The turbidity was assessed with a Sigrist Labscat device (Sigrist-photometer, Germany). The BGs were measured using a calcofluor method according to Kupetz et al. (2016a), and AXs were measured with a modified Douglas method based on Steiner et al. (2023). Statistical differences among the beers were assessed by Student's t distribution (p-value <0.05, JMP® Pro v. 17.0.0, SAS, USA)

### 2.2 Sensory analysis of beers

A human sensory panel determined the sensory characteristics of all the produced beers (n = 12). Before the main sensory evaluation, a sensory panel did not detect any off-flavor on the products through DLG (Deutsche Landwirtschafts-Gesellschaft e.V, German Agriculture Society) evaluation (supplementary data, Table A.2). On average, 15 panelists (4–5 female and 10–11 male per session, 25–40 years old) from the Chair of Brewing and Beverage Technology tasted the samples. The panelists take part in weekly training sessions to be able to internally rate beer samples. However, the *PF* and *mouthfeel* descriptors were only verbally explained to the panelists before every sensory session. The beer samples were evaluated in three independent sensory sessions (one-week timespan among sessions), where one replicate from each of the four beers (blank, barley, wheat, and oat) was presented in a random order in a three-digit coded plastic cup. Thus, only four samples were given per session to prevent sensory fatigue. A nose-clip was used during the whole session to prevent bias due to a difference in aroma compounds among the beers. The drinking temperature of the samples was 15 °C. The intensity and hedonic characteristics were evaluated in the same sensory session. The *PF* and *mouthfeel* descriptors were evaluated using an intensity scale from 0 to 7. PF *intensity* (*PFI*) describes how strong the sample is, while PF *quality* (*PFQ*) quantifies the pleasant level. On the other hand, *mouthfeel* descriptors (*watery* and *slimy*) may serve as *PFQ* indicators (Krebs et al., 2021). Hence, *watery* was defined as the lack of fullness (or body) of the liquid, and slimy as the unpleasant mucilage-like sensation in the mouth. Harmony, defined as the ratio between sweet and sour, was evaluated with a “just about right” scale where a 4 was set as a good balance (harmonic) sample. Higher values indicate that the sample is predominantly sweet, while the sourness is mainly abundant on lower values. After rating their intensities, the panelists were asked to rank the beers according to their *PF* and *mouthfeel* overall *preference* from lowest to highest. The intensity scores were averaged, and the statistical differences assessed with Student's t distribution (JMP® Pro v. 17.0.0). The overall preference differences were evaluated by critical absolute rank sum differences at a 5 % level of significance (Watts et al., 1989).

The produced beers had minor differences in ethanol content. Due to the familiarity of the sensory panel with beer products, their ethanol sensitivity might differ from what is reported in the literature. Therefore, an ethanol threshold determination of the sensory panel was done to decipher at which level the panelists are negatively influenced by a difference in ethanol content during the *PF* and *mouthfeel* sensory evaluation. A triangle test was set to determine the ethanol threshold using a commercial bottom-fermented NAB (0.5 vol%) as a matrix. The ethanol was spiked at two concentration levels (0.5 % and 1 % vol%). The samples were presented in a three-digit coded plastic cup containing the NAB and one spiking level. The panelists were asked to taste the samples (15 °C) while wearing a nose clip (to prevent any aroma deviations) and forcedly select the odd sample.

### 2.3 Isolation of beers' (non-)starch polysaccharides

The (N)SPs in all the beers were enzymatically isolated based on a literature method (Moreno Ravelo et al., 2024b). 100 mL of degassed beer (ultrasound for 5 min, Sonorex Super RK 255 H, Germany) was concentrated to 15 mL twice to generate a total volume of 30 mL of beer concentrate. Three 4 mL aliquots of beer concentrate were precipitated with 36 mL of ethanol and stored overnight at 4 °C. The supernatant was discarded after centrifugation (4 °C, 4000 g, 20 min, 55810R centrifuge, Eppendorf, Germany) followed by an ethanol wash (10 mL 95 % v/v). After discarding the supernatant, the pellet was dried for 24 h at 65 °C and re-dissolved with 4 mL sodium acetate buffer (0.1 M, pH 5.0, 65 °C, Sigma-Aldrich Bio Ultra, Germany) with sodium azide (0.025 % w/v, Carl Roth, Germany). Different enzymes were added to each aliquot to obtain different (N)SPs: 100 μL of Attenuzyme® Pro (Novozymes, Denmark) and 100 μL of lichenase (E-LICHN, Megazyme, Ireland) for AXs; 100 μL of Attenuzyme® Pro, 20 μL of endo-1,4-β-xylanase (E-XYLNP, Megazyme, Ireland), and 100 μL of β-ᴅ-xylosidase (E-BXSR, Megazyme, Ireland) for BGs; and 100 μL of lichenase, 20 μL of endo-1,4-β-xylanase, and 100 μL of β-ᴅ-xylosidase for DXs. After enzymatic hydrolysis (50 °C, 100 rpm, 24 h), each polysaccharide was further isolated by using a preparative size exclusion chromatography column (Hiload 26/600 Superdex 200 PG; Cytiva Europe GmbH, Germany). The eluent (50 % diluted phosphate buffer, pH 7.0, Honeywell Chemicals, Germany) was controlled with the Äkta Go device (Cytiva Europe GmbH, Germany). The hydrolyzed samples were centrifuged three times (5000 rpm, 5 min, 25 °C; Sigma 6k15 centrifuge, Sigma Laborzentrifugen, GmbH, Germany) to avoid an excess of particles contaminating the preparative column. 3 mL of the hydrolyzed samples were injected with a 10 mL Superloop™ (Cytiva Europe GmbH, Germany). After 90 mL of flow (2.6 mL/min) upon sample injection, 130 mL of sample was collected with the F9-R fraction collector (Cytiva Europe GmbH, Germany). It is important to mention that some proteins may remain in the extracts as they can interact with the polysaccharides and co-elute during the preparative size fractionation (Zielke et al., 2017). At last, the freeze-dried polysaccharides (Beta 1–8 LSCplus, Christ, Germany) were re-dissolved in 4 mL bi-distilled water and syringe filtered into vials (0.2 μm, PES 20/25, Chromafil, Macherey-Nagel, Germany). The purity of the extracts was not assessed. However, the complete enzymatic digestion of the of high molar mass polysaccharides in the matrix by using all the hydrolytic enzymes followed by their analysis by asymmetric flow-field flow fractionation coupled with a multi-angle light scattering and differential refractive index detectors (AF4-MALS-DRI) suggests that 80.3 % of the mass detected are the (N)SPs of interest.

### 2.4 Physical characterization of beers' (non-)starch polysaccharides isolates by AF4-MALS-DRI

All the isolated polysaccharides were independently fractionated by AF4, and their absolute molar mass was analyzed by coupling a MALS (DAWN HELEOS II, Wyatt Technology Europe, Germany) and a DRI (1260 series, Agilent Technologies, Germany) detectors. A long separation channel with trapezoidal geometry (tip-to-tip length of 26.5 cm, inlet width 2.1 cm, and outlet width 0.6 cm) was used for AF4 fractionation. A 10 kDa ultrafiltration regenerated cellulose membrane (Wyatt Technology Europe, Germany) was placed between the channel frit and the 350 μm spacer. The actual channel thickness was 225.8 ± 2.7 μm, which was calculated with the FFFHydRad v. 2.0 MATLAB extension (Lund University) based on BSA's retention time, the channel's dimensions, and separation conditions according to the literature (Litzén, 1993).

The fractionation in the AF4 channel was performed with a quaternary pump (1100 series, Agilent Technologies, Germany) controlled with the Eclipse 3+ (Wyatt Technology Europe, Germany). The mobile phase comprised 50 mM sodium nitrate (Emplura®, Merck, Germany) and 0.025 % w/v of sodium azide dissolved in bi-distilled water. The detector flow was 1 mL/min. A focus time of 3 min at 3 mL/min crossflow was set upon sample injection (25 μL) with an autosampler (1100 series, Agilent Technologies, Germany). A 0.1 μm spacer was placed between the pump and the autosampler to prevent oversized particles from entering the system. The sample eluted with a crossflow exponential decay with a half-life of 4 min, with a minimum crossflow of 0.1 mL/min during the rest of the elution. At last, the fractionated samples flowed through the detectors coupled in a series, first the MALS detector, followed by the DRI detector.

ASTRA software versions 6.1.2 and 6.1.7 were used to record and calculate the molar mass and size data, respectively (Wyatt Technology Europe, Germany). The molar mass ($M_{w}$) and size ($r_{rms}$) were obtained with the Berry fitting method using eleven scattering angles (34.8–142.5°) and a dn/dc of 0.146 for all the (N)SPs. The hydrodynamic radius ($r_{hyd}$) and the apparent density ($\rho_{app\;}$) were calculated with the FFFHydRad v. 2.0 Matlab extension as explained in detail somewhere else (Nilsson et al., 2006). To accomplish this, raw data from the Astra software was extracted, including the DRI and MALS signals, $M_{w}$, $r_{rms}$, and their respective elution times. The $r_{hyd}$ was calculated from the retention time of the analyte by following a numerical approach based on the fractionation settings (Hakansson et al., 2012; Magnusson et al., 2012). The $\rho_{app\;}$ was determined from the molar mass and the $r_{hyd}$ by assuming a homogenous mass distribution and spherical shape (Hakansson et al., 2012). Additionally, the $r_{rms}/r_{hyd}$ was also calculated and used as a conformational parameter, whose value dictates the physical state of the polysaccharide in solution: >2 a rod, 1.78 and 1.5 for random coiled, 1.73 and 1.23 for branched polysaccharides, 0.775 sphere, and <0.7 highly swollen molecule (micro-gel) (Nilsson, 2013).

The average response of the physical characteristics from each parameter of each beer sample was calculated. Statistical differences were evaluated with Student's t distribution (p-value <0.05, PCA, JMP® Pro v. 17.0.0). Additionally, the conformation characteristics per molar mass distribution plots for each (N)SPs is available in the supplementary data (Fig. A.1).

### 2.5 Soft tribology determination

The soft tribology of beer was assessed following a literature method with minor modifications (Moreno Ravelo et al., 2025). The surfaces used for the measurements, the devices, and the software to control them were acquired from Anton Paar GmbH (Germany). A steel ball (12.7 mm) and PDMS (Sylgard 184) pins were used to simulate the palate and tongue surfaces, respectively (Biegler et al., 2016). The T-PTD 200 tribology attachment with a ball-on-3 pins configuration was mounted to the MCR 502 WESP rheometer to determine friction (details below). The RheoCompass software (v. 1.30.1227) was used to control the system and record the data.

Before all the measurements, the motor adjustment and measurement of the system inertia were performed from a 1 mm contact point distance. 500 μL of degassed beer sample (by sonicating 100 mL at room temperature for 5 min) was added into the sample holder, which was set at 27 °C. The normal force was fixed at 6 N with a ramp-down logarithmical speed (1–10^−8^ m/s). The samples were always analyzed in three sequential runs, changing to a new degassed beer aliquot at the beginning of each run. The first two runs were used to equilibrate (running-in) the PDMS pins, and the third run was considered for the friction calculation. The PDMS pins were exchanged after these sequential runs. All the beer samples were measured in technical duplicates, reporting the standard deviation from the biological triplicate samples.

### 2.6 Statistical evaluation

MATLAB R2022b software (MathWorks, United States) was used to run the FFFHydRad v. 2.0 program. The average and standard deviation from the (N)SPs' chromatograms were calculated with the average multiple curves option (based on the same X, retention time) of OriginPro (2020) (v. 9.7.0.188., OriginLab Corporation, United States). Thus, the recorded AF4 data was reduced from 879 to 100 points without losing data integrity.

Principal component analysis (PCA, JMP® Pro v. 17.0.0) was done to explain the sensory results and to reduce the data dimensionality. For the former, the average values of all the descriptors of each biological beer triplicate (n = 3) were used. For the latter, PCA was independently done on all the parameters to reduce the dimensionality, with three principal components (PCs) per parameter. The relationship between the measured parameters from the (N)SPs to the *PF* and *mouthfeel* was analyzed by partial least squares regression (PLSR, JMP® Pro v. 17.0.0). The sensory descriptors were set as the dependent parameters (y-loadings) and PCs of soft tribology and AF4-MALS-DRI as the independent parameters (x-loadings). The average sensory values for each descriptor were used for PLSR. The centering, scaling, and standardized X options were enabled for the PLSR, which used the NIPALS method to build the model. The data was filtered from two PLSR models by removing all the parameters with a variable of importance (VIP) of less than 0.8 (supplementary data, Table A.3), followed by a third and final model (Coulon-Leroy et al., 2018). A leave-one-out cross validation method was used on the third model, the optimum number of latent factors was two based on the minimized root mean predicted residual sum squares (PRESS) of 1.25, which in turn comprised for a total variation of 80.75 % and 92.89 % in the x- and y-axis, respectively. As the PLSR was built with reduced data by PCA, the relevant loadings of each PC within the final PLSR can be observed in the supplementary data (Fig. A.2–A.5). The standardized coefficients from the final PLSR were extracted (supplementary data, Table A.4) to find the relationship between the sensory descriptors and the measured parameters with the aid of a two-way hierarchical clustering (JMP® Pro v. 17.0.0). The Ward method was used for clustering the data, which was standardized by column with the standardize robustly option enabled.

## 3 Results and discussion

### 3.1 Chemical analysis of the beers

The substitution of 25 % of standard malt with a low modified malts from different resources changed the chemical characteristics of the beers compared to the control (in Table 1). This is especially observed in the BGs concentration, from which the beer substituted with O39 malt presented a significantly higher concentration (*t*-test, p-value <0.05) followed by B39, the control, and W39. Other significant differences presented include the apparent extract, real extract, viscosity adj. to 8.6 %, soluble nitrogen, soluble nitrogen adj. to 12 %, and turbidity. Minor differences were observed among the rest of the measured characteristics.

**Table 1:** Chemical characteristics of brewed beers. Average chemical characteristics of beers brewed with 25 % of low modified malt grains (steeping degree 39 %). The letters represent a statistical difference calculated with *t*-test (n = 3, p-value <0.05). The error showed is the standard deviation calculated from the value of three independent beers replicates. B39: barley malted at 39 % steeping degree; O39: oat malted at 39 % steeping degree; W39: wheat malted at 39 % steeping degree.

Parameter	Unit	Control	B39	O39	W39
pH	[-]	4.5 ± 0.1a	4.5 ± 0.1a	4.5 ± 0a	4.4 ± 0.1a
Alcohol	[vol%]	4.6 ± 0.6a	4.9 ± 0.1a	4.5 ± 0.1a	4.6 ± 0.1a
Original Gravity	[wt%]	10.7 ± 1.3a	11.2 ± 0.2a	10.5 ± 0.3a	11.1 ± 0.1a
Apparent Extract	[wt%]	1.9 ± 0.3b	2 ± 0b	2 ± 0b	2.5 ± 0a
Real Extract	[wt%]	3.6 ± 0.5b	3.7 ± 0ab	3.6 ± 0.1b	4.1 ± 0a
Viscosity	[mPa·s]	1.607 ± 0.127a	1.628 ± 0.044a	1.664 ± 0.026a	1.698 ± 0.02a
Viscosity Adj. to 8.6 %	[mPa·s]	3.398 ± 0.342ab	3.417 ± 0.296ab	3.8 ± 0.109a	3.302 ± 0.134b
Apparent Attenuation	[%]	81.97 ± 0.73a	82.46 ± 0.44a	80.91 ± 0.15b	77.88 ± 0.45c
Real Attenuation	[%]	67.44 ± 0.41a	67.89 ± 0.38a	66.56 ± 0.15b	64.23 ± 0.37c
Final degree of attenuation	[%]	82.2 ± 0.7b	83.9 ± 0.5a	82.3 ± 0.5b	79 ± 0.4c
β-Glucan	[mg/L]	199.4 ± 42.7c	251.7 ± 13b	361.2 ± 14.6a	193 ± 23.7c
AX	[mg/L]	731.6 ± 51.1a	620.7 ± 170.7a	666.7 ± 28.5a	799.1 ± 65.7a
Soluble Nitrogen	[mg/L]	602.9 ± 75.6b	709.8 ± 12.8a	607.8 ± 20.9b	617.6 ± 12.2b
Soluble Nitrogen Adj. To 12 %	[mg/L]	663.5 ± 15.1bc	755.1 ± 6.6a	680.6 ± 10.6b	645.8 ± 11.7c
Iso-alpha acids	[mg/l]	15 ± 1.8a	15.2 ± 2.7a	18 ± 0.3a	18 ± 1.8a
Bitter units	[EBC]	16.9 ± 1.4a	18.4 ± 1a	17.5 ± 0.7a	18.6 ± 2.5a
Total Polyphenols	[mg/L]	115.6 ± 30a	135 ± 17.1a	108.2 ± 9.9a	105 ± 3.8a
Turbidity 90°	[EBC Formazine]	1.6 ± 0.1b	1.6 ± 0.4b	2.4 ± 0.1a	2 ± 0.4ab
Turbidity 25°	[EBC Formazine]	0.5 ± 0.2b	0.5 ± 0.4b	1.4 ± 0.5a	1 ± 0.3ab

Previous research suggests the importance of the beer's chemical characteristics when evaluating the sensory perception of cereal-based beverages as different parameters (e.g., extract or BGs content) could influence the sensory response (Krebs et al., 2021; Langstaff et al., 1991b; Kupetz et al., 2016b). This effect was also observed when raw materials with different physical characteristics were used, from which it was suggested that the BGs and DXs concentration ratio was relevant (Moreno Ravelo et al., 2024a). Therefore, the beers were designed to prevent, as much as possible, any chemical or physical bias outside of the possible molecular perception provided by the (N)SPs. Among the resulting samples, the biggest average differences were the original gravity and alcohol content with 0.7 wt% and 0.4 vol%, respectively. The threshold detection of ethanol in water is 0.66 %; however, the matrix used influences this threshold, being 2.9 % in water and 6 % in beer (Guttman et al., 2019). Since our sensory panel may be more sensitive due to the in-house training sessions, the level (threshold) at which an ethanol difference is perceived might be different. Thus, this parameter was assessed on the panelists at two levels, perceiving only a difference with a 1 % addition of ethanol (n = 11, p-value <0.05), which is higher than the alcohol difference present in the beer samples (0.4 vol%, Table 1). Regarding original gravity, the beer samples showed a difference of 0.7 wt%. According to the literature, a difference of 0.050 of original gravity (1.2 wt%) is necessary to detect a change based on sensory characteristics such as *PF* (Brown and Clapperton, 1978). Thus, it can be suggested that the differences in alcohol content (0.4 vol%) and original gravity (0.7 wt%) among the beer samples do not influence the *PF* and *mouthfeel* perception. However, other parameters, such as soluble nitrogen, may affect the sensory perception because they were not adjusted to a similar level with the current experimental setup.

### 3.2 Palate fullness and mouthfeel sensory evaluation of beers

**Fig. 1:**
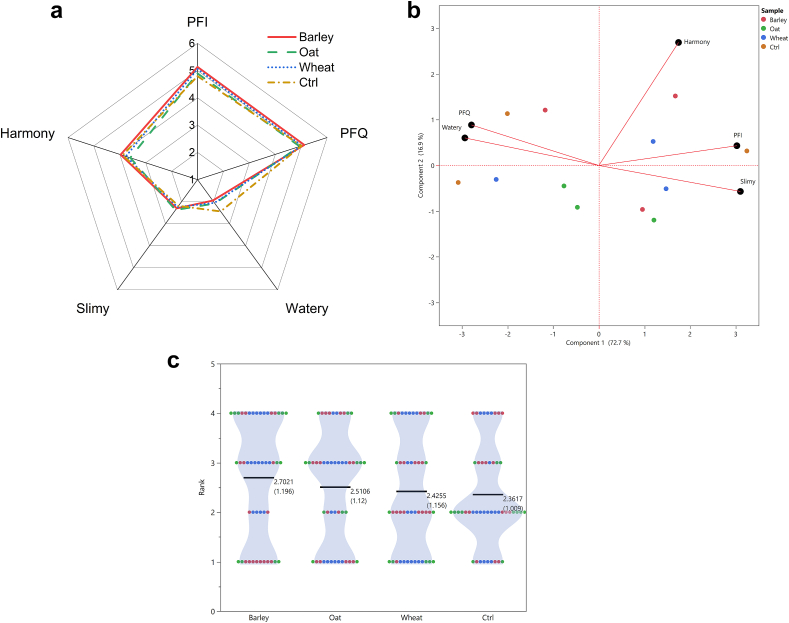
Sensory characteristics of the beers. Sensory results of beers produced with 25 % subsitution of low-modified malt grains (39 % steeping degree) in biological triplicates (n = 3). One beer from each sample group was independently tasted on three sensory sessions, leading to all the samples being tasted once. The sensory results based on intensity scale are shown in the spider diagram (a) and PCA (b). The preference ranking test (c) results are depicted as scattered density plot; the average value and standard deviation are also depicted. Each color represents a sample within the triplicate beers in (b) and (c). PFI: palate fullness intensity; PFQ: palate fullness quality.

A human sensory panel analyzed the different descriptors for *PF* (*intensity* and *quality*) and *mouthfeel* (*watery* and *slimy*) as well as the harmony. The average results (n = 12) may be observed in Fig. 1a (overall averages in the supplementary data, Table A.5). The beer substituted with B39 (5.1 ± 0.3) and W39 (5.1 ± 0.3) malts were rated with the highest *PFI*, followed by O39 (4.9 ± 0.2). The (unsubstituted) control was the most *watery* sample (2.4 ± 0.7), suggesting this was the weakest beer according to the panelists. This descriptor was similarly evaluated on the beers brewed with O39 and W39 malts (2.1 ± 0.3), while the lowest rated sample was the beer substituted with B39 malt (2 ± 0.4). The O39 beer was evaluated as the *slimiest* (2.4 ± 0.2), followed by B39 (2.3 ± 0.2), W39 (2.2 ± 0.4), and control (2.2 ± 0.7). However, no statistical differences were observed among the samples (Student's t, p-value <0.05).

A PCA was performed on the sensory evaluation, covering 89.6 % of the data with the first two PCs (Fig. 1b; loading values in the supplementary data, Table A.6). The first PC was composed of *PFI* and *sliminess* on the positive loading, while *PFQ* and *watery* on the negative. *PFI* was negatively correlated to *watery* and *PFQ* to *slimy*, which is in accordance to the literature (Krebs et al., 2021; Moreno Ravelo et al., 2024a). Harmony was present in the second PC. The deviation of the beer sensory evaluation is observed when analyzing the score positions in the bi-plot. The beer samples substituted with O39 were evaluated more consistently as they showed similar scores, while the other samples had only two samples with similar scores.

Regarding the ranking preference test, the beer sample substituted with B39 malt was on average the most preferred followed by the O39 and W39 beer samples, but no statistical differences were observed by the sum ranking test (Fig. 1c). According to the intensity scale, the B39 beer was rated with the highest *PFI*, and a moderate *PFQ* and *sliminess*. The least preferred sample was the control, which was rated by the sensory panel as the highest *watery*.

Previous research showed that non-harmonic NAB (<3, sour dominant) may negatively influence the sensory perception from high molar mass polysaccharides when evaluating the *PF* and *mouthfeel* (Moreno Ravelo et al., 2023). All the beer samples had a minor sour balance as the harmony range was 3.6–4.0 according to the sensory panel. A possible reason behind this might be related to a masking effect of sourness due to the alcohol content in beer compared to NAB (Martin and Pangborn, 1970). Thus, the lack of sourness of the beers allowed the panelists to perceive the high molar mass (N)SPs during the beer sensory evaluation.

### 3.3 Molar mass and conformation of beer's (N)SPs

Previous work suggests a change in the physical characteristics of the (N)SPs at the laboratory level (Moreno Ravelo et al., 2025), but the presence of these varied structures needed to be confirmed on a larger scale. Thus, the molar mass and conformation of (N)SPs, isolated from the beers, were analyzed by AF4-MALS-DRI (Moreno Ravelo et al., 2024b). The average values are shown in Table 2. The polydispersity (PDI) nature of the (N)SPs can be better interpreted when plotting the conformation behavior based on the molar mass, which can be found in the supplementary data, with some key features being discussed in the text (Fig. A.1).

**Table 2:** Molar mass and conformation of starch and non-starch polysaccharides in bottom fermented beers. A different letter indicates a statistical difference by Student's t (p-value <0.05).

			Control	B39	O39	W39
Arabinoxylans	$M_{w}$	[kDa]	176.4 ± 36.7a	181.4 ± 36.5a	147.1 ± 2.8a	138.1 ± 9.2a
	PDI	[-]	1.8 ± 0.2a	2 ± 0.4a	1.7 ± 0a	1.6 ± 0.1a
	$r_{rms}$	[nm]	20.8 ± 12.2a	10.2 ± 4.3ab	8 ± 0.6b	8.9 ± 0.6ab
	$r_{hyd}$	[nm]	5.8 ± 0.2a	6.1 ± 0.3a	6.1 ± 0.1a	6.1 ± 0.2a
	$\rho_{app\;hyd}$	[kg/m3]	502.2 ± 49.1a	424.6 ± 30.4b	444.8 ± 9.8b	413.6 ± 8.7b
	$r_{rms}/r_{hyd}$	[-]	2.6 ± 1.3a	1.4 ± 0.5ab	1.2 ± 0.1b	1.2 ± 0.1b
	Mass det.	[μg]	173.7 ± 5.6a	180.9 ± 9.1a	186.9 ± 11a	185.3 ± 5.1a
β-glucans	$M_{w}$	[kDa]	120.6 ± 11b	131.4 ± 17.1ab	143.1 ± 10.8a	112.8 ± 4.3b
	PDI	[-]	1.3 ± 0.1b	1.4 ± 0.2ab	1.6 ± 0.1a	1.3 ± 0b
	$r_{rms}$	[nm]	8.3 ± 0ab	9.8 ± 3.5a	9.7 ± 3.2ab	5.1 ± 1.3b
	$r_{hyd}$	[nm]	5.2 ± 0.2c	5.5 ± 0.2b	5.8 ± 0.1a	5.2 ± 0.1bc
	$\rho_{app\;hyd}$	[kg/m3]	554.3 ± 38.3a	519.5 ± 21.5a	467.1 ± 25.9b	506.3 ± 15ab
	$r_{rms}/r_{hyd}$	[-]	1.3 ± 0.1ab	1.4 ± 0.2a	1.4 ± 0.3a	0.9 ± 0.1b
	Mass det.	[μg]	154.9 ± 3.5a	169.5 ± 11.3a	169.5 ± 15.2a	155 ± 6.5a
Dextrins	$M_{w}$	[kDa]	1800.8 ± 149.2a	1483.5 ± 363.9a	1860.1 ± 1006a	1838.5 ± 252.1a
	PDI	[-]	21 ± 1.9a	16.6 ± 4.2a	25.6 ± 11.7a	25.4 ± 1.2a
	$r_{rms}$	[nm]	20 ± 7.9a	21.5 ± 5.3a	14.5 ± 3.4a	16.6 ± 3.4a
	$r_{hyd}$	[nm]	9.4 ± 0.8ab	9.3 ± 0.7ab	10.5 ± 0.6a	8.4 ± 0.2b
	$\rho_{app\;hyd}$	[kg/m3]	332.9 ± 67a	321.6 ± 42.5a	251.3 ± 37.4a	301.4 ± 47.7a
	$r_{rms}/r_{hyd}$	[-]	1.6 ± 0.3a	1.8 ± 0.4a	1.4 ± 0.3a	1.6 ± 0.3a
	Mass det.	[μg]	34.9 ± 9.9c	75.9 ± 11.2b	88.3 ± 9.1ab	93.3 ± 0.7a
(N-)SP ratio	AXs	[%]	47.8	42.4	42	42.7
	BGs	[%]	42.6	39.8	38.1	35.8
	DXs	[%]	9.6	17.8	19.9	21.5

Average physical characteristics of beers brewed with 25 % of low modified malt grains (steeping degree 39 %). The letters represent a statistical difference calculated with *t*-test (n = 3, p-value <0.05). The error showed is the standard deviation calculated from the value of three independent beers replicates. B: barley; O: oats; W: wheat; AXs: arabinoxylans; BGs: *β*-glucans; DXs: dextrins; $M_{w}$: weight average molar mass; PDI: polydispersity; $r_{\text{rms}}$: root mean square radius; $r_{\text{hyd}}$: hydrodynamic radius; $\rho_{\text{app}\;\text{hyd}}$: apparent density calculated from hydrodynamic radius; $r_{\text{rms}}/r_{\text{hyd}}$: conformation ratio; Mass det.: mass detected in the AF4 (>10 kDa).

The substitution of standard malt with low-modified malt modified the (N)SPs' physical characteristics (molar mass and conformation) when compared to the control. Regarding (N)SPs, the AXs’ $M_{w}$ (138.1 ± 9.2 kDa) and apparent density based on hydrodynamic radius ($\rho_{app\;hyd}$, 413.6 ± 8.7 kg/m^3^) of W39 beer sample decreased compared to the control (176.4 ± 36.7 kDa and 502.2 ± 49.1 kg/m^3^), while the $M_{w}$ of BGs was significantly higher when the B39 (131.4 ± 17.1 kDa, Student's t, p-value <0.05) and O39 (143.1 ± 10.8 kDa, Student's t, p-value <0.05) malt was used. In comparison to the control (1800.8 ± 149.2 kDa), substituting with B39 malt shifted the DXs' $M_{w}$ to a lower value (1483.5 ± 363.9 kDa), while O39 (1860.1 ± 1006 kDa) and W39 (1838.5 ± 252.1 kDa) presented a slight increase of $M_{w}$.

Besides the brewing process, the molar mass responses can be influenced by raw material source (Moreno Ravelo et al., 2025), its modification characteristics (Moreno Ravelo et al., 2024a), and malt modification (Krebs et al., 2020). The molar mass increase of BGs, as observed in the beers substituted with B39 and O39 malts, was expected as less BGs degradation occurs with a lower modification during malting, generating higher molar masses (Moreno Ravelo et al., 2024a; Henry, 1988). A similar behavior was also documented for DXs in laboratory-produced worts (Moreno Ravelo et al., 2025). Respecting AXs, substituting 30 % of the malt bill with unmalted rye increased the concentration of high molar mass AXs (determined by GC-MS) (Langenaeken et al., 2020). However, only the beer produced with B39 agreed with this behavior, which can be attributed to the intrinsic characteristics of AXs in each grain (He et al., 2021; Izydorczyk et al., 2009).

The conformation ratio ($r_{rms}/r_{hyd}$) values indicate the presence of a wide variety of structures in all the (N)SPs. AXs and BGs were present as swollen polysaccharides (<0.5), micro-gel (<0.7), sphere (0.775), random coiled (1.5, 1.78), and rod molecules (>2) (Nilsson, 2013). Additionally, AXs seemed to be present as aggregates on all the samples as their $\rho_{app\;hyd}$ increases in the high molar mass region (>1000 kDa, Fig. A.1). However, the $r_{rms}/r_{hyd}$ on the same high molar mass region changed, thus hindering the confirmation of the aggregates (Håkansson et al., 2012). The aggregation was not observed with the BGs on all the beer samples. The $r_{rms}/r_{hyd}$ values of DXs suggest the presence of elongated ($r_{rms}/r_{hyd}$ = >2) and branched structures ($r_{rms}/r_{hyd}$ = 1.23 and 1.73 for hyper and randomly branched, respectively (Nilsson, 2013)). This can be confirmed when observing their conformation when plotting the $r_{rms}/r_{hyd}$ based on the molar mass (Fig. A.1), from which the $\rho_{app\;hyd}$ increase around the $r_{rms}/r_{hyd}$ value of 1.23 confirms the presence of branches in DXs (Nilsson, 2013).

The DXs’ conformation behavior differs from the literature because the elongated structures observed in the beer samples were not so broadly present in the laboratory-produced wort (Moreno Ravelo et al., 2025). This difference might be due to the differences in raw material and process (mashing). Rübsam et al. demonstrated that the initial mashing temperature (Rübsam et al., 2013a) and the starch source (Rübsam et al., 2013b) influence the resulting molar mass in beer. Additionally, altering the mashing temperature modifies the active enzymes and, thus, shifts the product's physical characteristics. The laboratory-produced wort was mashed with an isothermal process at 63 °C, in which the β-amylase is mainly active producing maltose (Back et al., 2019). Differently, the brewing process involved two saccharification steps, activating the β-amylase (62 °C) and α-amylase (72 °C), the latter enzyme producing megalosaccharides and oligosaccharides as the main products. As α-amylase was only active during the brewing process, the higher presence of elongated structures and branches in the beer than in the wort is feasible. It is important to remark that comparing wort and beer physical characteristics is possible based on the assumption that these characteristics minorly change after the boiling step (Krebs et al., 2020). Additionally, the intrinsic physical characteristics of the standard barley malt used as a control may influence the results, but their sole characterization was not performed.

The substitution of low-modified malt also influenced the mass detected of the (N)SPs. For example, the DXs’ mass detected increased in all the beers, being statistically different from the control (*t*-test, p-value <0.05). Additionally, the substitution of low-modified malt changed the ratio of the mass detected of the (N)SPs (>10 kDa), especially with the DXs because their ratio increased in all the beers compared to the control. An interesting effect is observed on this parameter when analyzing the mass detected of BGs. A similar mass detected increased compared to the control was observed on the beers substituted with B39 (169.5 ± 11.3) and O39 (169.5 ± 15.2) malts, but their conformation behavior differed. The beer substituted with B39 malt showed a significantly higher $\rho_{app\;hyd}$ than the one substituted with the O39 malt (519.5 ± 21.5 kg/m^3^ versus 467.1 ± 25.9 kg/m^3^; p-value <0.05, Student's t). This confirms that substituting standard barley malt with a low modified malt (steeping degree as parameter) varies the concentration and conformation of the (N)SPs.

**Fig. 2:**
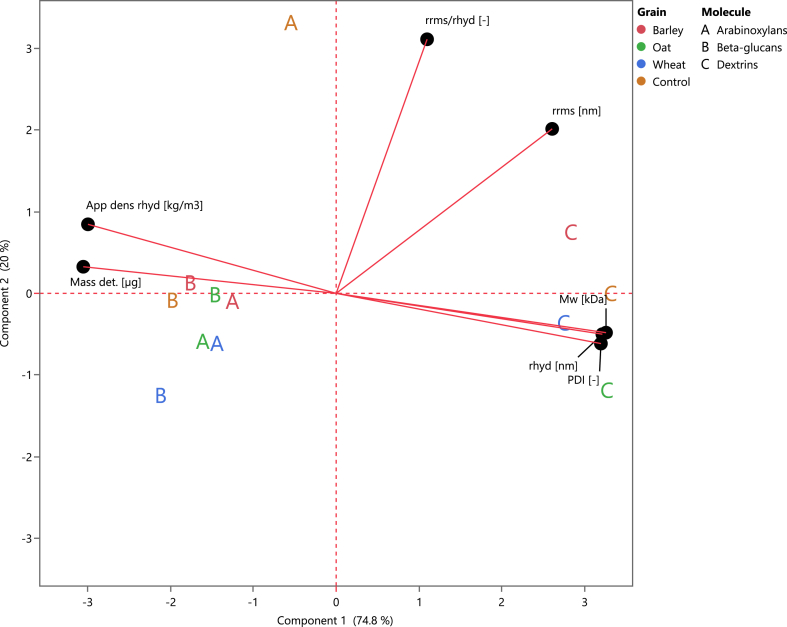
The principal component analysis (biplot) of the molecular charachterisitcs of (N)SPs. The average molar mass and conformation of (N)SPs extracted from beer produced with 25 % subsitution of low-modified malt grains (39 % steeping degree) from different sources (n = 3). Each letter represents different (N)SPs. A: arabinoxylans; B: β-glucans; C: dextrins; $M_{w}$: weight average molar mass; PDI: polydispersity; $r_{rms}$: root mean square radius; App dens rhyd: hydrodynamic radius; $\rho_{app\;hyd}$: apparent density calculated from hydrodynamic radius; $r_{rms}/r_{hyd}$: conformation ratio; Mass det.: mass detected.

The main physical characteristics of the samples are summarized by a PCA (Fig. 2). Two PCs explain 94.8 % of the data (loading values in the supplementary data, Table A.4). The positive axis of the PC1 contains $M_{w}$, $r_{hyd}$, and PDI, while $\rho_{app\;hyd}$ and mass detected are placed in the negative loading of this component, which indicate a negative correlation among these parameters. $r_{rms}/r_{hyd}$ and $r_{rms}$ are part of PC2. Overall, the DXs were characterized for presenting high molar masses and PDI, and a big size. Within the DXs, O39 had on average the most compact structures as they were placed the furthest away from the $r_{rms}/r_{hyd}$ loading (PC2). Regarding the (N)SPs, AXs and BGs presented similar characteristics, displaying high densities and mass detected.

In summary, different molar mass and conformation values were found among the (N)SPs. The magnitude of the measured parameters was in a similar range to previous works (Moreno Ravelo et al., 2025), but the beer sample substituted with low modified malt showed an increase of $r_{rms}/r_{hyd}$. This difference was attributed to using different mashing temperatures during the wort production. Therefore, the different conformation structures in the beer samples can be confirmed.

### 3.4 Soft tribology (friction) of beers

**Fig. 3:**
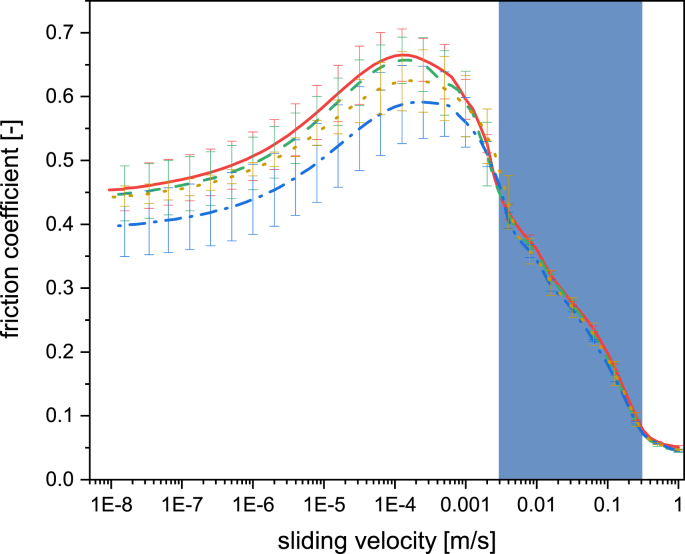
Stribeck curves of beer samples. Each color represents a beers produced with 25 % subsitution of low-modified malt grains (39 % steeping degree) from different sources: barley beer by a full red line, oat beer by a dashed green line, and wheat beer by a dash dot line. The yellow dots represent the control beer (unsubstituted beer). The error bar depicts the standard deviation of each beer sample being independently analyzed in triplicate (n = 3). The blue rectangle represents the in-mouth velocities obtained from tongue velocities reported from Peng et al. (2000).

The resulting FC is displayed in Stribeck curves (Fig. 3). Only the mixed (1–≈0.008 m/s) and a combination of boundary and static regimes (≈0.008–1 E^−8^ m/s) were present when assessing the beers. After starting the measurements at high velocity (1 m/s), the gap between the measuring surfaces decreases, causing the FC to arise. Nevertheless, the FC of all the samples was similar in the mixed regime until a sliding velocity of around 0.001 m/s. A further decrease in speed provokes a transition from the mixed to the boundary regime, inferred from the FC's peak apex. This transition point (maximum FC) was slightly different among the beer samples. The W39 and control samples reached this point around 0.008 and 0.009 m/s, respectively, while a velocity of 0.0095 m/s was needed for both B39 and O39 samples. Despite the lack of a “breakaway point” depicting the transition between the static and the boundary regimes (Pondicherry et al., 2018), a sliding velocity of 4.5 E^−7^ m/s was suggested as the transition velocity between these regimes when a ramp-down velocity is applied to our current device settings (Moreno Ravelo et al., 2025).

The B39 sample showed the highest maximum FC (0.668 ± 0.039), followed by the O39 (0.659 ± 0.038), the control (0.626 ± 0.048), and W39 beer samples (0.596 ± 0.056). Compared to the laboratory worts derived from the same low modified malted grains (Moreno Ravelo et al., 2025), the maximum FC values are comparable, but the magnitude of response among the samples changed (e.g., the wort produced with W39 depicted the highest maximum FC while it was the lowest in the beers). Interestingly, the FC in the static/boundary regime of the O39 beer (≈0.45 at 1 E^−8^ m/s) drastically changed to higher values compared to the wort sample derived from the same O39 malted grain (≈0.1 at 1 E^−8^ m/s). These results suggest a loss in lubricity due to a lack of polymer adsorption in the boundary regime, causing the FC to increase (Stokes et al., 2011; You et al., 2020).

### 3.5 Contribution of (N)SPs physical characteristics and soft tribology to the sensory perception

A PLSR was used to analyze the relationship between sensory perception and the analytical parameters. The intention of this strategy was only to model the samples of this work with the sole purpose of interpreting the relationship between sensory descriptors and physical/tribology characteristics (exploratory) rather than to build a predictive model. Achieving robust predictive capability was not the goal of this work, and doing so would require a substantially larger dataset. Nevertheless, this strategy was chosen based on its capacity to filter through numerous collinear x-variables as present in chromatography and tribology data (Wold et al., 2001).

**Fig. 4:**
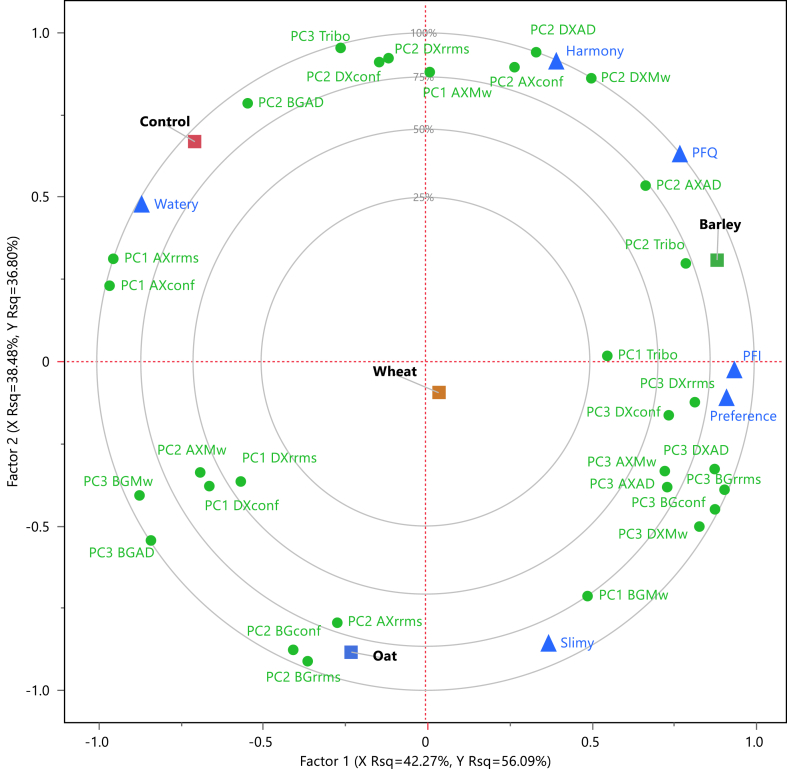
Correlation loading plot analyzed by PLS. This graph shows the relationship between physical (molar mass, conformation, and tribology, (x) and the sensory responses (y). PC: principal component; AX: arabinoxylans; BG: β-glucans; DX: dextrins; Tribo: tribology; Mw: molar mass; AD: apparent density; rrms: root mean square radius; conf: conformation ratio.

Two latent factors were selected, containing factor 1 a variation explained of 56.09 % in the y-axis and 42.27 % in the x-axis. The sensory descriptors that are mainly contained in this factor are *PFI*, *preference*, and *watery*. Meanwhile, 36.80 % and 38.48 % of variation is explained in factor 2 for the y-axis and x-axis data, respectively, with *slimy* and *harmony* as the primary sensory descriptors. *PFQ* was the only sensory descriptor that was equally contained in both factors. The correlation loading plots summarize all the data by displaying the composition of the samples and their relationship to the sensory description (Fig. 4). For example, the B39 beer was characterized by a high *PFQ*, *PFI*, and sensory preference while also showing relationships with the apparent density of AXs (PC2 AXAD), tribology (PC2 Tribo), and DXs rms (PC3 DXrrms) and $r_{rms}/r_{hyd}$ (PC3 DXconf).

**Fig. 5:**
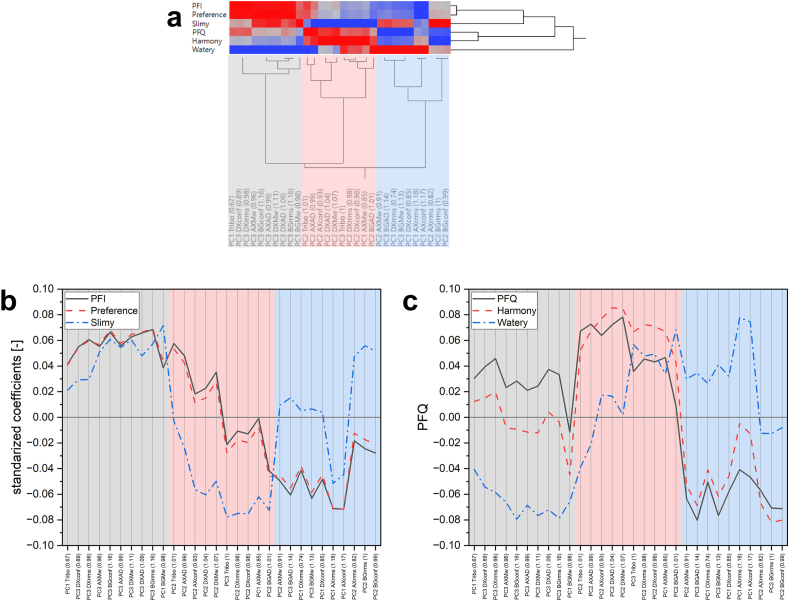
Parameters influencing the sensory characteristics of beers. (a) Two-way hierarchical clustering between the sensory characteristics and measured parameters based on PLS standardized coefficients. The sensory descriptors were separated according to their clustering behavior: (b) cluster 1 (*palate fullness intensity*, *PFI*; *preference*) and cluster 2 (*slimy*); (c) cluster 3 *(palate fullness quality*, PFQ; and *harmony*) cluster 4 (*watery*). PC: principal component; AX: arabinoxylans; BG: β-glucans; DX: dextrins; Tribo: tribology; Mw: molar mass; AD: apparent density; rrms: root mean square radius; conf: conformation ratio.

The standardized coefficients from the PLSR were adopted for a two-way hierarchical clustering as they describe the magnitude and direction of the relationship between the sensory descriptors and the measured parameters (Fig. 5). Three clusters were defined for the measured parameters. In contrast, four types of PLSR coefficient behaviors were observed among the sensory descriptors (Fig. 5a). The physical characteristics and FC showed multiple positive relationships to the sensory characteristics. First, the relationship between FC and sensory response will be discussed.

The differences in the order of magnitude among the positive standard coefficients suggest that the FC and the sensory descriptors present distinct correlations depending on the sliding velocity (tribology's PC 1–3). For example, the PC1 and PC2 of tribology present a positive correlation to *PFI, slimy,* and *preference (*Fig. 5b) and PC3 to *watery, harmony, and PFQ* (Fig. 5c). Based on the PCA's loading plot (Fig. A.2), the PC2 was comprised by the sliding velocities from the mixed regime (1 and 0.4 m/s) and the in-mouth velocity of 0.32 m/s, while the PC1 by the rest of the in-mouth velocities excluding 0.0032 m/s, which was relevant for PC3. Based on these results, it can be concluded that most of the FC from the in-mouth velocities (0.32–0.0063 m/s), high velocities corresponding to the mixed regime (1–0.32 m/s), the boundary/static regimes, and the maximum FC positively correlate to *PFI*, *slimy* and panelist *preference,* whilst only the in-mouth velocity at 0.0032 m/s may serve as a *watery, PFQ,* and *harmony* indicators.

The previous analysis suggests that most in-mouth sliding velocities relevant to oral processing showed a different positive relationship to the sensory characteristics depending on their magnitude. Additionally, the magnitude differences in the PLSR coefficients from the PC1/PC2 tribology between *PFI* and *slimy* sensory descriptors suggest further differences when analyzing each velocity independently. Thus, a correlation table showing the relationship between sensory descriptors and FC was done (Table 3). Positive correlations were observed depending on the sliding velocity, as suggested by the PLSR model. This is more evident when comparing the *PFI* and *slimy* descriptors, where high sliding velocities (1–0.063 m/s) led to higher correlations to *PFI* but negative correlations to *slimy*. Interestingly, *slimy* showed positive correlations upon a sliding velocity decrease, while *PFI* had the opposite behavior. Based on these observations, it can be hypothesized that *PFI* and *slimy* descriptors may be perceived in the mouth at different stages during oral processing because the correlation to FC varies according to the sliding velocity (Peng et al., 2000). Although *PF* and *mouthfeel* are generally considered in the same umbrella term in the brewing industry (Langstaff and Lewis, 1993), Krebs et al. demonstrated that a human sensory panel is capable of independently recognizing these descriptors by spiking different polysaccharides into NAB (Krebs et al., 2019). However, further research is needed to confirm this hypothesis.

**Table 3:**
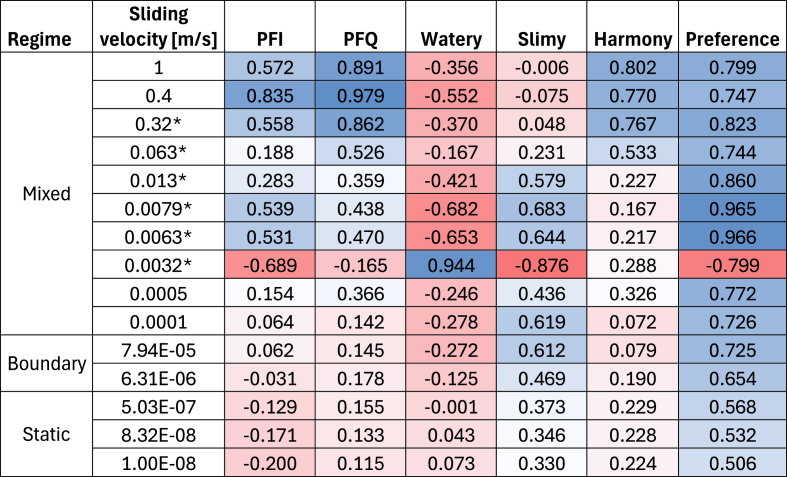
Correlation coefficients between sensory descriptors and friction coefficients. The color scheme represents the intensity of the correlation from the 1 to −1 scale. ∗In-mouth velocity based on Peng et al., 2000 (Peng et al., 2000).

Similarly to the soft tribology data, different (N)SPs showed a positive relationship to the sensory descriptors based on the positive standardized coefficients. The standardized coefficients in the first cluster (Fig. 5b) depict the parameters that mainly influence *PFI*, sensory *preference*, and *slimy*. Among these parameters, DXs' physical characteristics (PC3 DXconf and PC3 DXrrms) stand out as distinctive parameters influencing the *PFI* and sensory *preference,* which were also suggested in the correlation loading plot (Fig. 4). However, these two parameters also present high standardized coefficients in *PFQ* (>0.04, Fig. 5c), which in turn presented its distinctive parameters along with the *harmony* (PC2 AXconf, PC2 DXAD, PC2 DXMw). When analyzing the PCA loadings from the PC3 DXconf and PC3 DXrrms (Fig A.4), the relevant retention time is the same for both parameters (4–7 min). Consequently, the range of PC DXconf was 1.6–3.9 (10–90 percentile; P10–P90), which corresponded to rod-like and branched molecules (Nilsson, 2013), while the P10–P90 for $r_{rms}$ was 28.9–12.5 nm.

The positive relationship between *PFI* and DXs in beer has been reported in the literature, being their concentration and molar mass important (Rübsam et al., 2013a). Additionally, this work suggests the shape (conformation) of DXs as an important parameter for *PFI*, *PFQ*, and sensory *preference*. Although the $r_{rms}/r_{hyd}$ values were not statistically different when comparing the control and the beer substituted with the B39 malt, the combination of $r_{rms}/r_{hyd}$ and a statistically higher mass detected (>10 kDa, p-value <0.5, Student's t) observed in the B39 beer (75.9 ± 11.2) suggested a higher amount of branched DXs compared to the control sample (34.9 ± 9.9). Thus, the distinctive standardized coefficients for PC3 DXconf and the greater amount of branched DXs observed in the beer sample substituted with B39 malt confirms the relevance of branched DXs for the higher *PFI, PFQ,* and panelist *preference*.

The multivariate analysis also suggests a distinctive relationship between AXs (PC1 AXrrms and PC1 AXconf) and the w*atery* mouthfeel descriptor. This relationship can be inferred from Fig. 5c as positive coefficients are observed in cluster 3 (blue), from which the highest standardized coefficients are primarily present on parameters derived from AXs. Similar to DXs, the conformation of AXs also seems to be relevant, but the loadings from the PCA analysis (Fig. A.3) shows a broader $r_{rms}/r_{hyd}$ and $r_{rms}$ P10–P90 range of 0.7–2.5 and 10.1–43.5 nm, respectively.

Finally, the *slimy* and panelist *preference* coefficients were positive in clusters 1 and 3, presenting distinctive values in the third cluster, in which the characteristics of (N)SPs are mainly present. Among all the standardized coefficients positively influencing the *slimy* response in the beers, the higher positive responses were found in the BGs' physical characteristics (cluster 1, PC1 BGMW; cluster 2, PC2 BGrrms and PC2 BGconf). Thus, a clear relationship between slimy and BGs can be suggested.

The sensory influence of the (N)SPs has been suggested in the literature. The positive relationship between the physical characteristics of BGs and the *slimy* descriptor is in accordance with the literature because the BGs are known to influence this descriptor in cereal-based beverages (Krebs et al., 2019; Moreno Ravelo et al., 2024a). Additionally, this work suggests that the conformation of BGs is relevant, especially in the PC2 BGconf P10–P90 range that corresponds to the sphere and highly swollen (micro-gel) structures ($r_{rms}/r_{hyd}$ of 0.7–0.9) (Nilsson, 2013). However, the positive relationship between *watery* and AXs was surprising because the high molecular weight AXs (DP 35+, ≈6.3 kDa) have been reported to contribute to the *PF* or body increase of beer (Langenaeken et al., 2020). A possible explanation for this is a lack of low molecular weight DXs on the beers substituted with low modified malt because their combination with high molecular weight AXs (avDP 82–113) is needed to improve the *PF* of NABs (Michiels et al., 2025). The literature can further support this as using of low modified malts produces fewer amount of low molar mass DXs on laboratory-produced worts (Moreno Ravelo et al., 2025). However, the reported low molecular weight DXs in the literature (≈6.3 kDa) might not be observed in the AF4 separation channel due to the ultrafiltration membrane (10 kDa), hindering the detection of these DXs in the substituted beer samples by the approach used in this research. Although it can be argued that not all the polysaccharides under the membrane threshold would permeate it (Guo et al., 2023), it is impossible to determine the extent to which these polysaccharides remained in the separation channel and are analyzed.

## 4 Conclusions

The *PF* and *mouthfeel* of beer were studied from the physical characteristics of (N)SPs and oral processing (drinking) perspectives. Multiple grain sources (barley, oat, and wheat) modified at a lower steeping degree (39 %) were used to vary the physical characteristics of the (N)SPs by substituting 25 % of the (standard barley malt) grain bill. The (N)SPs from the produced beers, including unsubstituted control, were isolated and analyzed by AF4-MALS-DRI. Finally, the soft tribology behavior of the beers was assessed as a tool to further characterize the beer samples outside of the traditional human sensory analysis. Among the several parameters showing positive relationships to the sensory attributes, the conformation of the (N)SPs, assessed by $r_{rms}/r_{hyd}$, was a distinctive physical characteristic. The $r_{rms}/r_{hyd}$ in DXs suggested that rod-like and branched structures were among the main factors influencing beer's *PFI, PFQ,* and panelist *preference*. Additionally, the higher *PFI* observed in the B39 beer was attributed to a higher presence of different conformations (branching) than the control. Regarding the (N)SPs, the $r_{rms}/r_{hyd}$ of BGs hinted that sphere and highly swollen structures influenced the *slimy mouthfeel* descriptors. Meanwhile, physical characteristics of AXs ($r_{rms}/r_{hyd}$ and $r_{rms}$) were correlated to the *watery mouthfeel* descriptor. Soft tribology in the sliding velocity range of 1–0.0063 m/s showed positive correlations to the analyzed sensory descriptors assessed by a human sensory panel. Furthermore, as *PF* descriptors showed a positive correlation at different (high) sliding velocities compared to the *mouthfeel* descriptors, it can be suggested that these sensory characteristics are perceived at different stages during oral processing, which also suggests the independence of each attribute. However, further research is needed to confirm this theory.

These results underline the relevance of the physical characteristics of (N)SPs towards sensory responses. However, it is important to mention that the sensory impact of proteins was not considered for this study, which could also contribute to the *PF* of the beers. Nevertheless, this research may aid the brewing industry in improving the sensory characteristics of beer and NAB since the (N)SPs can be modulated through the malting process, which also influences the soft tribology. Hence, the generation of improved raw materials naturally enriched with the desired (N)SPs fractions to enhance the relevant sensory characteristics of the product is possible.

## Ethical statement

All procedures performed in studies involving human participants were in accordance with the ethical standards of the institutional and national research committee and with the 1964 Helsinki declaration and its later amendments or comparable ethical standards.

## CRediT authorship contribution statement

Rolando Cesar Moreno Ravelo: Conceptualization, Formal analysis, Investigation, Methodology, Project administration, Validation, Visualization, Writing – Original draft.

Christoph Neugrodda: Resources, Writing – review & editing.

Martina Gastl: Conceptualization, Resources, Supervision, Writing – review & editing.

Thomas Becker: Supervision, Writing – review & editing.

## Formatting of funding sources

This research did not receive any specific grant from funding agencies in the public, commercial, or not-profit sectors.

## Declaration of competing interest

The authors declare that they have no known competing financial interests or personal relationships that could have appeared to influence the work reported in this paper.

## Data Availability

Data will be made available on request.
