# Supplementary material for: Contribution of (non-)starch polysaccharides to distinctive sensory perception in beer – Significance of their physical and friction characteristics
Source: Curr Res Food Sci. 2025 Jun 18;11:101118. doi: 10.1016/j.crfs.2025.101118 (PMC12273455; doi:10.1016/j.crfs.2025.101118)
Supplement: Multimedia component 1 [file mmc1.docx]

**Supplementary data**

**Contribution of (non-)starch polysaccharides to distinctive sensory perception in beer – Significance of their physical and friction characteristics**

Rolando Cesar Moreno Ravelo*^1^, Christoph Neugrodda^1^, Martina Gastl^2^, Thomas Becker^1^

^1^Technical University of Munich, Institute of Brewing and Beverage Technology.

Group Raw Material Based Brewing and Beverage Technology, Weihenstephaner Steig 20, 85354, Freising, Germany.

^2^Technical University Munich, Research Center Weihenstephan for Brewing and Food Quality, Alte Akademie 3, 85354, Freising, Germany

Tel.: +49 8161 71-5170. Fax: +49 8161 71-4181

*Corresponding author. E-mail address: [rolando.moreno-ravelo@tum.de](mailto:rolando.moreno-ravelo@tum.de)

ORCID: Rolando César Moreno Ravelo 0000-0002-1278-870X

**Table A.1. Chemical characteristics of malt grains from different sources.** Standard malt analysis of malt from different sources (iso 65 °C, n = 3). Table values extracted from supplementary material of [1].

| **Sample** | **S.D.** | **pH-Wert** | **Extract** | **Viscosity** | **Color** | **β-Glucan** | **Soluble Nitrogen** | **FAN** | **FDA** | **Friability** |
| --- | --- | --- | --- | --- | --- | --- | --- | --- | --- | --- |
|  | **[%]** |  | **[%w/v]** | **[mPa·s]** | **[EBC]** | **[mg/L]** | **[mg/L]** | **[mg/L]** | **[%]** | **[%]** |
| Barley | 39 | 6.1 ± 0 | 81.2 ± 0.6 | 0.981 ± 0.005 | 6.3 ± 0.1 | 265.1 ± 63.9 | 846.5 ± 14.3 | 160.2 ± 3.7 | 86.7 ± 0.4 | 66.2 ± 0.1 |
| Barley | 45 | 6.1 ± 0 | 81.4 ± 1.1 | 0.9 ± 0.005 | 6.7 ± 0.2 | 42.5 ± 1.7 | 894.2 ± 7.5 | 180.4 ± 2 | 89.7 ± 0.1 | 86.1 ± 1.2 |
| Wheat | 39 | 6.2 ± 0 | 84.8 ± 0.2 | 1.212 ± 0.004 | 9.6 ± 0.3 | 63 ± 8.7 | 602.2 ± 9.9 | 73.4 ± 0.9 | 80.1 ± 0.1 |  |
| Wheat | 45 | 6.1 ± 0 | 85.5 ± 0.4 | 1.18 ± 0.004 | 11.7 ± 0.4 | 36 ± 5.4 | 742.6 ± 3.5 | 94.4 ± 1.8 | 81.5 ± 0.4 |  |
| Oat | 39 | 6 ± 0 | 59.9 ± 1.8 | 0.938 ± 0.012 | 23.9 ± 2.1 | 368.5 ± 5.2 | 472.8 ± 9.9 | 79 ± 1.3 |  |  |
| Oat | 45 | 5.8 ± 0 | 60.5 ± 0.5 | 0.938 ± 0.008 | 20.3 ± 2.1 | 297 ± 9.6 | 517.2 ± 11.1 | 102 ± 1.9 |  |  |

**Table A2. Sensory results based on the DLG scale.** This scheme evaluates the beer quality from based the beer type (bottom-fermented lager beer) being 5 the highest score available.

|  | **Aroma** | **Taste** | **Palate fullness** | **Carbonation** | **Quality of bitterness** |
| --- | --- | --- | --- | --- | --- |
| Control | 4.7 ± 0.1 | 4.4 ± 0.1 | 4.7 ± 0.1 | 4.9 ± 0.1 | 4.8 ± 0 |
| B39 | 4.4 ± 0.3 | 4.3 ± 0.4 | 4.8 ± 0.1 | 4.8 ± 0.2 | 4.7 ± 0.1 |
| O39 | 4.4 ± 0.2 | 4.3 ± 0.1 | 4.7 ± 0.1 | 4.8 ± 0.2 | 4.6 ± 0 |
| W39 | 4.6 ± 0.2 | 4.3 ± 0.2 | 4.8 ± 0.1 | 4.9 ± 0.2 | 4.7 ± 0.1 |

**Fig. A.1. Conformation parameters and molar mass plots of different (N-)SP isolated from beers.** The apparent density calculated from hydrodynamic radius ($\rho_{app hyd}$) is depicted as full lines, while the dashed lines the conformation ratio (${r_{rms}}/{r_{hyd}}$ Different colors represent the grain used for brewing: yellow for control, red barley, green oats, and blue wheat.

**Table A.3. Variable of importance (VIP) values.** VIP values calculated from sequential partial least squares (PLS) each containing less independent parameters based on VIP values lower than 0.8.

| **1st PLS** | | **2nd PLS** | | **3rd PLS** | |
| --- | --- | --- | --- | --- | --- |
| **X** | **VIP** | **X** | **VIP** | **X** | **VIP** |
| PC1 Tribo | 0.8264 | PC1 Tribo | 0.8317 | PC1 Tribo | 0.6701 |
| PC2 Tribo | 1.1344 | PC2 Tribo | 1.0371 | PC2 Tribo | 1.0072 |
| PC3 Tribo | 1.0797 | PC3 Tribo | 0.9654 | PC3 Tribo | 1.0046 |
| PC1 AXMw | 0.93 | PC1 AXMw | 0.8581 | PC1 AXMw | 0.8494 |
| PC2 AXMw | 1.0401 | PC2 AXMw | 0.9721 | PC2 AXMw | 0.9094 |
| PC3 AXMw | 1.0903 | PC3 AXMw | 1.0088 | PC3 AXMw | 0.9614 |
| PC1 AXrrms | 1.3007 | PC1 AXrrms | 1.1526 | PC1 AXrrms | 1.1793 |
| PC2 AXrrms | 0.9114 | PC2 AXrrms | 0.8511 | PC2 AXrrms | 0.8203 |
| PC3 AXrrms | 0.783 | PC1 AXAD | 0.7841 | PC2 AXAD | 0.9934 |
| PC1 AXrhyd | 0.6418 | PC2 AXAD | 1.0168 | PC3 AXAD | 0.9888 |
| PC1 AXAD | 0.8127 | PC3 AXAD | 1.0249 | PC1 AXconf | 1.169 |
| PC2 AXAD | 1.1102 | PC1 AXconf | 1.1471 | PC2 AXconf | 0.9341 |
| PC3 AXAD | 1.1147 | PC2 AXconf | 0.9186 | PC2 DXMw | 1.0667 |
| PC1 AXconf | 1.2926 | PC2 DXMw | 1.0285 | PC3 DXMw | 1.1086 |
| PC2 AXconf | 1.0132 | PC3 DXMw | 1.0886 | PC1 DXrrms | 0.7386 |
| PC3 AXconf | 0.6619 | PC1 DXrrms | 0.8502 | PC2 DXrrms | 0.9848 |
| PC1 DXMw | 0.5589 | PC2 DXrrms | 0.9711 | PC3 DXrrms | 0.9785 |
| PC2 DXMw | 1.1531 | PC3 DXrrms | 1.0165 | PC2 DXAD | 1.0403 |
| PC3 DXMw | 1.2221 | PC2 DXAD | 0.9984 | PC3 DXAD | 1.0915 |
| PC1 DXrrms | 0.8737 | PC3 DXAD | 1.0864 | PC1 DXconf | 0.8524 |
| PC2 DXrrms | 1.0682 | PC1 DXconf | 0.9212 | PC2 DXconf | 0.9802 |
| PC3 DXrrms | 1.1095 | PC2 DXconf | 0.97 | PC3 DXconf | 0.8863 |
| PC DXrhyd | 0.6437 | PC3 DXconf | 0.9538 | PC1 BGMw | 0.9763 |
| PC1 DXAD | 0.643 | PC1 BGMw | 0.9929 | PC3 BGMw | 1.1268 |
| PC2 DXAD | 1.1181 | PC3 BGMw | 1.1133 | PC2 BGrrms | 1.0026 |
| PC3 DXAD | 1.2132 | PC2 BGrrms | 0.9692 | PC3 BGrrms | 1.1642 |
| PC1 DXconf | 0.9791 | PC3 BGrrms | 1.1403 | PC2 BGAD | 1.0052 |
| PC2 DXconf | 1.0651 | PC2 BGAD | 0.9853 | PC3 BGAD | 1.1367 |
| PC3 DXconf | 1.0209 | PC3 BGAD | 1.1081 | PC2 BGconf | 0.9902 |
| PC1 BGMw | 1.0819 | PC2 BGconf | 0.9638 | PC3 BGconf | 1.1555 |
| PC2 BGMw | 0.6428 | PC3 BGconf | 1.1312 |  |  |
| PC3 BGMw | 1.2457 |  |  |  |  |
| PC1 BGrrms | 0.5644 |  |  |  |  |
| PC2 BGrrms | 1.0813 |  |  |  |  |
| PC3 BGrrms | 1.2836 |  |  |  |  |
| PC BGrhyd | 0.6418 |  |  |  |  |
| PC1 BGAD | 0.613 |  |  |  |  |
| PC2 BGAD | 1.0976 |  |  |  |  |
| PC3 BGAD | 1.247 |  |  |  |  |
| PC1 BGconf | 0.6052 |  |  |  |  |
| PC2 BGconf | 1.0723 |  |  |  |  |
| PC3 BGconf | 1.2726 |  |  |  |  |

**Fig. A.2. PCA loadings from soft tribology analysis.** Blue color depicts the tongue velocity according to Peng, et al. (2000) [2].

**Fig. A.3. PCA loadings from arabinoxylans molar mass and conformation analysis.** PC: principal component; Mw: weight average molar mass; r_rms_: root-mean-square radius; AD: apparent density calculated from the hydrodynamic radius; conf: conformation ratio, r_rms_/r_hyd_

**Fig A.4. PCA loadings from dextrins molar mass and conformation analysis.** PC: principal component; Mw: weight average molar mass; r_rms_: root-mean-square radius; AD: apparent density calculated from the hydrodynamic radius; conf: conformation ratio, r_rms_/r_hyd_

**Fig. A.5. PCA loadings from β-glucans molar mass and conformation analysis.** PC: principal component; Mw: weight average molar mass; r_rms_: root-mean-square radius; AD: apparent density calculated from the hydrodynamic radius; conf: conformation ratio, r_rms_/r_hyd_

**Table A.4. Standardized PLS coefficients.** Three clusters were obtained after performing a hierarchical clustering analysis of the coefficients. The number in parenthesis represents the VIP values from the PLS model.

| **Term** | **PFI** | **PFQ** | **Watery** | **Slimy** | **Harmony** | **Preference** |
| --- | --- | --- | --- | --- | --- | --- |
| Cluster | 1 | 3 | 4 | 2 | 3 | 1 |
| PC1 Tribo (0.67) | 0.0415 | 0.0305 | -0.0407 | 0.0213 | 0.0124 | 0.0409 |
| PC2 Tribo (1.01) | 0.0576 | 0.0673 | -0.0392 | -0.0027 | 0.0524 | 0.0536 |
| PC3 Tribo (1) | -0.0213 | 0.0358 | 0.0568 | -0.0778 | 0.0666 | -0.0277 |
| PC1 AXMw (0.85) | -0.0008 | 0.0467 | 0.0338 | -0.0619 | 0.0668 | -0.007 |
| PC2 AXMw (0.91) | -0.0496 | -0.0634 | 0.0298 | 0.0096 | -0.053 | -0.0454 |
| PC3 AXMw (0.96) | 0.0552 | 0.0232 | -0.0662 | 0.0509 | -0.0082 | 0.0567 |
| PC1 AXrrms (1.18) | -0.0711 | -0.0408 | 0.0778 | -0.0515 | -0.0049 | -0.0717 |
| PC2 AXrrms (0.82) | -0.0183 | -0.0572 | -0.0125 | 0.0473 | -0.0674 | -0.0124 |
| PC2 AXAD (0.99) | 0.048 | 0.0727 | -0.021 | -0.0239 | 0.0673 | 0.0425 |
| PC3 AXAD (0.99) | 0.0558 | 0.0211 | -0.0685 | 0.0545 | -0.0115 | 0.0576 |
| PC1 AXconf (1.17) | -0.0717 | -0.0467 | 0.0745 | -0.0446 | -0.0128 | -0.0715 |
| PC2 AXconf (0.93) | 0.0182 | 0.0638 | 0.0174 | -0.0562 | 0.0769 | 0.0113 |
| PC2 DXMw (1.07) | 0.0353 | 0.0782 | 0.0018 | -0.0498 | 0.0846 | 0.028 |
| PC3 DXMw (1.11) | 0.0628 | 0.0244 | -0.0767 | 0.0605 | -0.0121 | 0.0648 |
| PC1 DXrrms (0.74) | -0.0413 | -0.0505 | 0.0264 | 0.0051 | -0.0409 | -0.038 |
| PC2 DXrrms (0.98) | -0.0108 | 0.0455 | 0.0478 | -0.075 | 0.0725 | -0.0176 |
| PC3 DXrrms (0.98) | 0.0607 | 0.0459 | -0.0586 | 0.0294 | 0.02 | 0.0597 |
| PC2 DXAD (1.04) | 0.0227 | 0.0722 | 0.0165 | -0.0605 | 0.0855 | 0.0151 |
| PC3 DXAD (1.09) | 0.0658 | 0.0374 | -0.0721 | 0.048 | 0.0041 | 0.0663 |
| PC1 DXconf (0.85) | -0.0483 | -0.0576 | 0.0319 | 0.004 | -0.0457 | -0.0447 |
| PC2 DXconf (0.98) | -0.0129 | 0.0433 | 0.0494 | -0.0751 | 0.0709 | -0.0196 |
| PC3 DXconf (0.89) | 0.0548 | 0.0394 | -0.0544 | 0.0293 | 0.015 | 0.0542 |
| PC1 BGMw (0.98) | 0.0386 | -0.0115 | -0.0656 | 0.0716 | -0.045 | 0.0433 |
| PC3 BGMw (1.13) | -0.0633 | -0.0767 | 0.0411 | 0.0066 | -0.0616 | -0.0585 |
| PC2 BGrrms (1) | -0.0245 | -0.0708 | -0.0127 | 0.0559 | -0.082 | -0.0173 |
| PC3 BGrrms (1.16) | 0.0685 | 0.0334 | -0.0789 | 0.0571 | -0.0036 | 0.0697 |
| PC2 BGAD (1.01) | -0.0418 | 0.0086 | 0.0684 | -0.0725 | 0.0433 | -0.0463 |
| PC3 BGAD (1.14) | -0.0606 | -0.0803 | 0.0346 | 0.0152 | -0.0687 | -0.0552 |
| PC2 BGconf (0.99) | -0.0279 | -0.0712 | -0.008 | 0.0516 | -0.0802 | -0.0209 |
| PC3 BGconf (1.16) | 0.0665 | 0.0283 | -0.0795 | 0.0608 | -0.0093 | 0.0682 |

**Table A.5. Raw data of sensory evaluation of beers.** Average sensory characteristics of beers produced with 25% substitution of low modified (39% steeping degree) malted grains from different sources. The error shows the standard deviation calculated from the independent sensory evaluation of the beer replicates (n = 3). B39: barley malted at 39% steeping degree; O39: oat malted at 39% steeping degree; W39: wheat malted at 39% steeping degree; PFI: palate fullness intensity; PFQ: palate fullness quality.

| **Beer** | **PFI** | **PFQ** | **Watery** | **Slimy** | **Harmony** |
| --- | --- | --- | --- | --- | --- |
| Control | 4.8 ± 0.4 | 5 ± 0.5 | 2.4 ± 0.7 | 2.2 ± 0.7 | 3.9 ± 0.4 |
| B39 | 5.1 ± 0.3 | 5.1 ± 0.2 | 2 ± 0.4 | 2.3 ± 0.2 | 4 ± 0.4 |
| O39 | 4.9 ± 0.2 | 4.9 ± 0.2 | 2.1 ± 0.3 | 2.4 ± 0.2 | 3.6 ± 0 |
| W39 | 5.1 ± 0.3 | 5 ± 0.3 | 2.1 ± 0.3 | 2.2 ± 0.4 | 3.8 ± 0.3 |

**Table A.6. Loading matrix of the beer´s sensory characteristics.**

|  | **Prin1** | **Prin2** | **Prin3** | **Prin4** | **Prin5** |
| --- | --- | --- | --- | --- | --- |
| PFI | 0.93257 | 0.13318 | 0.28408 | -0.17460 | 0.03717 |
| PFQ | -0.86065 | 0.27331 | 0.41419 | 0.10756 | 0.03829 |
| Watery | -0.90514 | 0.18584 | -0.36529 | -0.10031 | 0.05185 |
| Slimy | 0.95636 | -0.17532 | -0.17304 | 0.14625 | 0.05745 |
| Harmony | 0.53841 | 0.83005 | -0.13672 | 0.04595 | -0.01804 |

**Table A.7. Loading matrix of the average responses of the starch and non-starch polysaccharides isolated from the beers.** Mw: weight average molar mass; r_rms_: root-mean-square radius; r_hyd_; hydrodynamic radius; App dens rhyd: apparent density calculated from the hydrodynamic radius; r_rms_/r_hyd_: conformation ratio; PDI: polydispersity (Mw/Mn); Mass det.: mass determined by concentration detector.

|  | **Prin1** | **Prin2** | **Prin3** | **Prin4** | **Prin5** | **Prin6** | **Prin7** |
| --- | --- | --- | --- | --- | --- | --- | --- |
| Mw [kDa] | 0.98329 | -0.14499 | -0.05450 | 0.09315 | -0.01220 | 0.01606 | 0.00811 |
| r_rms_ [nm] | 0.78706 | 0.60690 | -0.02551 | -0.07905 | -0.06564 | 0.03144 | -0.00253 |
| r_hyd_ [nm] | 0.96994 | -0.15062 | 0.09529 | -0.11013 | 0.12332 | 0.01046 | -0.00053 |
| App dens r_hyd_ [kg/m3] | -0.90436 | 0.25475 | -0.32951 | 0.05695 | 0.07017 | 0.02191 | -0.00032 |
| r_rms_/r_hyd_ [-] | 0.33127 | 0.93741 | 0.07382 | 0.06120 | 0.04146 | -0.02457 | 0.00164 |
| PDI [-] | 0.96468 | -0.18520 | -0.00374 | 0.18695 | 0.00920 | 0.00179 | -0.00607 |
| Mass det. [µg] | -0.92146 | 0.09822 | 0.36638 | 0.07779 | 0.01639 | 0.02653 | 0.00049 |

**References**

[1] Moreno Ravelo, R.C., Gastl, M., Becker, T., Relationship between physical characteristics of cereal polysaccharides and soft tribology–The importance of grain source and malting modification, Food Science and Nutrition 13(1) (2025) e4699.

[2] Peng, C.-L., Jost-Brinkmann, P.-G., Miethke, R.-R., Lin, C.-T., Ultrasonographic Measurement of Tongue Movement During Swallowing, Journal of Ultrasound in Medicine 19(1) (2000) 15-20.
